# The Regenerative Marriage Between High-Density Platelet-Rich Plasma and Adipose Tissue

**DOI:** 10.3390/ijms26052154

**Published:** 2025-02-27

**Authors:** Peter A. Everts, Luga Podesta, José Fabio Lana, George Shapiro, Rafael Barnabé Domingues, Andre van Zundert, Robert W. Alexander

**Affiliations:** 1 Medical School (GBCS), The University of Queensland, Brisbane, QLD 4006, Australia; a.vanzundert@uq.edu.au; 2 Center for Collaborative Research, Zeo Scientifix, Inc., NOVA Southeastern University, Fort Lauderdale, FL 33328, USA; gshapiro@zeoscientifix.com; 3 Medical School, Max Planck University Center (UniMAX), Indaiatuba 13343-060, SP, Brazil; josefabiolana@gmail.com (J.F.L.); dr.rafael@birm.com.br (R.B.D.); 4 Regenerative Medicine Group, Orthoregen International Course, Indaiatuba 13334-170, SP, Brazil; 5 Bluetail Medical Group and Podesta Orthopedic Sports Medicine, Naples, FL 34109, USA; lugamd@aol.com; 6 Orlando College of Osteopathic Medicine, Orlando, FL 34787, USA; 7 Clinical Research, Anna Vitória Lana Institute (IAVL), Indaiatuba 13334-170, SP, Brazil; 8 Royal Brisbane Clinical Unit, Faculty of Medicine, The University of Queensland, Brisbane, QLD 4072, Australia; 9 Regenevita Biocellular Aesthetic and Reconstructive Surgery, Cranio-Maxillofacial Surgery, Regenerative Medicine and Wound Healing, Hamilton, MT 5998840, USA; rwamd1914@gmail.com; 10 Department of Surgery and Maxillofacial Surgery, University of Washington, Seattle, WA 988104, USA

**Keywords:** high-density platelet-rich plasma, tissue stromal vascular fraction, adipose-derived mesenchymal stem cells, autologous platelet exosomes, tissue repair

## Abstract

The use of autologous biological preparations (ABPs) and their combinations fills the void in healthcare treatment options that exists between surgical procedures, like plastic reconstructive, cosmetic, and orthopedic surgeries; non-surgical musculoskeletal biological procedures; and current pharmaceutical treatments. ABPs, including high-density platelet-rich plasma (HD-PRP), bone marrow aspirate concentrates (BMACs), and adipose tissue preparations, with their unique stromal vascular fractions (SVFs), can play important roles in tissue regeneration and repair processes. They can be easily and safely prepared at the point of care. Healthcare professionals can employ ABPs to mimic the classical wound healing cascade, initiate the angiogenesis cascade, and induce tissue regenerative pathways, aiming to restore the integrity and function of damaged tissues. In this review, we will address combining autologous HD-PRP with adipose tissue, in particular the tissue stromal vascular fraction (t-SVF), as we believe that this biocellular combination demonstrates a synergistic effect, where the HD-PRP constituents enhance the regenerative potential of t-SVF and its adipose-derived mesenchymal stem cells (AD-MSCs) and pericytes, leading to improved functional tissue repair, tissue regeneration, and wound healing in variety of clinical applications. We will address some relevant platelet bio-physiological aspects, since these properties contribute to the synergistic effects of combining HD-PRP with t-SVF, promoting overall better outcomes in chronic inflammatory conditions, soft tissue repair, and tissue rejuvenation.

## 1. Introduction

Regenerative medicine, a specialized field of interventional and non-surgical medicine, often addresses issues not solvable by surgery or medications. There is a significant need for new treatment options for degenerated and traumatized tissues, as well as difficult-to-heal acute and chronic wounds. Regenerative procedures have gained significant attention in recent years due to the need for methods to restore and regenerate missing tissues and organs.

Autologous biomaterials, such as autologous bone grafts, are known for their ability to induce bone formation and generate new bone. However, their limited availability, the need for a second surgical site, and patient discomfort have restricted the use of these biomaterials, prompting the search for other effective alternatives [1]. Autologous biomaterials for regenerative medicine applications can be categorized into classes based on their primary biological source. These classes include bone-derived materials (e.g., bone grafts), cartilage-derived materials (e.g., chondrocytes), adipose- and bone marrow-derived mesenchymal stem cells, blood-derived products (e.g., platelets, plasma), skin-derived fibroblasts, and tissue-derived extracellular matrix (ECM) components. Each class possesses distinct chemical, biological, and physical properties that influence their suitability for specific tissue regeneration applications [2].

Tissue repair, regeneration, and wound healing constitute highly intricate and dynamic processes, influenced by a variety of pathological conditions, including chronic recalcitrant wounds, damaged tissue structures, and degenerative tissues. These processes are dependent upon an array of local and systemic multicellular biological activities, which encompass critical cell signaling mechanisms.

Autologous biological preparations, which can be prepared at the point of care, following strict aseptic techniques during tissue collection and the various processing steps to avoid adverse events [3], present significant promise for the promotion of tissue healing and repair in a natural manner [4,5]. The application of these preparations leverage the body’s inherent healing capabilities, thereby enhancing the repair process in a targeted and efficient fashion. Blood-derived and mesenchymal stem and stromal degenerative tissues-based preparations are crucial in tissue repair and wound healing processes within complex biological microenvironments [6].

Generally, HD-PRP and t-SVF biological preparations are increasingly used to support chronic wound healing and tissue repair by delivering autologous biological cells [7,8].

This review aims to provide a comprehensive overview of the nuances of HD-PRP preparations as a stand-alone treatment, and we will address the synergy between HD-PRP and t-SVF when both biologics are combined as a therapeutic preparation.

## 2. Healing Cascades

**Figure 1:**
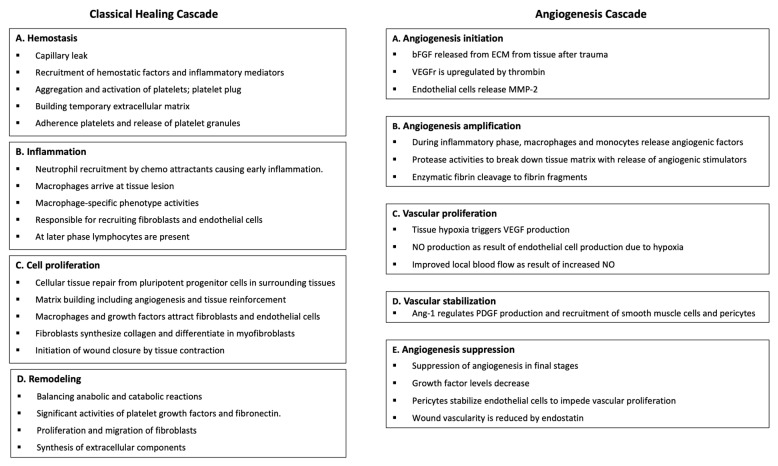
Overview of classical and angiogenesis healing cascades.

Both the classical and angiogenesis healing pathways consist of distinct phases with an overlap of cellular activities, and they work in conjunction to ensure a coordinated and efficient healing and repair process, as shown in Figure 1.

The traditional healing cascade consists of distinct phases, each characterized by specific cellular activities [9]. Briefly, during the initial phase, hemostasis, platelets aggregate at tissue injury sites, stimulating blood clotting while forming a fibrinous network. Platelets then regulate the healing process by secreting their granular content and releasing many PGFs, cytokines, and molecules that initiate tissue repair [10]. The angiogenesis cascade, though less studied, adds complexity to the wound healing process as it involves the recruitment, differentiation, and proliferation of endothelial cells [11]. Ultimately, new blood vessels are formed from preexisting ones, which is crucial for effective tissue repair, regeneration, and wound healing [12]. In both healing cascades, the cellular and acellular components of HD-PRP, as well as the constituents of adipose tissue (t-SVF), play pivotal roles in restoring or regenerating tissues. The platelet and t-SVF cellular and acellular components stimulate tissue repair and regeneration by promoting cell proliferation, cell migration, immunomodulation, and angiogenesis. When combined, HD-PRP and t-SVF work synergistically to facilitate effective functional tissue restoration and regeneration.

## 3. HD-PRP Definition

PRP can be defined as a centrifugated and processed liquid fraction of harvested fresh peripheral blood with a platelet concentration above the baseline value [13]. HD-PRP can be characterized as a heterogenous and complex composition of multicellular components, like platelet growth factors, chemokines, and other cytokines, in a small volume of plasma, with a platelet concentration greater than 1 × 10^9^/mL [13,14]. These natural proteins and cytokines are present in “normal” biological proportions and play important roles in cell proliferation, chemotaxis, cell differentiation, and angiogenesis [15].

## 4. A Brief Overview of Platelets

Platelets are small, anucleate, discoid blood cells, typically measuring 1–3 micrometers, with an in vivo half-life of 7–10 days. In adults, the average platelet count ranges from 150 to 400.000 per microliter of circulating blood [16]. Platelets are synthesized in the red bone marrow from megakaryocytes, which are their hematopoietic progenitor cells, by pinching off from these cells. Once synthesized, platelets are released into the peripheral circulation in a resting state and on a continuous basis [17]. They play a crucial role in hemostasis by aggregating at the site of blood vessel injury to form a clot, preventing excessive bleeding. Furthermore, platelets are actively involved in inflammation and immune responses, maintaining a dynamic balance of platelet production and clearance in the body [18]. Additionally, platelets contain numerous growth factors, chemokines, cytokines, and other molecules that are essential for tissue repair and regeneration [15].

The underlying scientific rationale for PRP therapy is that an injection of concentrated platelets at tissue sites may initiate repair through the release of numerous biologically active factors in the platelet storage granules, adhesion proteins, and transcription factors. These components are responsible for initiating restorative pathways. In any PRP formulation, platelets are the primary cells, and their activation leads to the secretion of these bioactive molecules [19].

The release of concentrated platelet biological components from HD-PRP can enhance tissue regeneration, promote cell proliferation, and accelerate the healing process by stimulating the body’s natural repair mechanisms.

## 5. PRP Variability and Its Consequences

Regrettably, significant variability in PRP platelet concentrations and formulations among currently available PRP devices has resulted in inconsistent platelet dosing regimens and cellular characterization, leading to less beneficial patient outcomes [20,21,22,23]. The argument presented is that a “one-size-fits-all” approach to PRP orthobiological preparations and applications should be replaced with more nuanced and transformative approaches [24]. These advances include the adoption of algorithms to determine cell dosing strategies specific to the pathoanatomic problem, as well as the use of physiologically different PRP bioformulations specific to the different tissues and pathologies being treated in the same procedure. This necessitates an unyielding awareness and understanding of the large variability in biocellular and orthobiological products currently on the market regarding cell type, quality, and quantity, and application volumes needed to achieve appropriate dosing. The physiological variability in orthobiological preparations and specific bioformulations regarding their efficacy in immunomodulation, angiogenesis, pain downregulation, and tissue repair has been noted in clinical studies [15]. Recently, we described recent advances in platelet–leukocyte interactions to provide a better understanding of PRP’s cellular behavior in various tissue types, like ligaments, menisci, and cartilage conditions, improving mechano-metabolic conditions, restoring musculoskeletal MSK function after injury and counteracting tissue degeneration and aging processes [24]. It is only fair to assume that within the same functional unit (e.g., knee joint), different tissue structures and pathologies would respond differently to different biological treatments because many interconnecting tissue structures are often involved in different pathologies. As a best practice, different PRP formulations should be used simultaneously in the same patient to treat different tissue-specific and pathoanatomic conditions. This approach aims to optimize the tissue structure-dependent healing response, ultimately leading to faster and better functional repair. For example, in patients with chronic wounds, different PRP formulations should be used based on the individual conditions of the wound bed and wound edges [4].

### 5.1. PRP Classification

Various PRP bioformulations, terminologies, and product descriptions have been introduced to practitioners over the years [25,26,27,28,29,30,31,32]. However, the lack of standardization and classification of PRP devices has led to the introduction of approximately 40 considerably different PRP devices to the market, with inconsistent cell capture rates and varying platelet and non-platelet cellular constituents [33].

Buffy-coat PRP devices and plasma-based PRP devices differ greatly. The more refined, often double-spin, buffy-coat PRP preparations have significantly more platelets available in the prepared PRP when compared to plasma-based PRP devices. Furthermore, buffy-coat devices in general process higher whole-blood volumes, and they have the ability to capture leukocytic cells as well. These PRP devices are considered more flexible in the utilization of different PRP preparation protocols and can therefore be more effective in creating positive patient outcomes. Furthermore, PRP preparations can be categorized as pure PRP, leukocyte-poor PRP (LP-PRP), or leukocyte-rich PRP (LR-PRP) [34].

In plasma-based PRP procedures, a “PRP-like” suspension is prepared, excluding erythrocytes and often leukocytes. Similarly, platelet-rich fibrin (PRF) plasma-based preparations primarily consist of plasma [35]. This plasma-based PRP product is a plasma protein matrix that can contain processed platelets and other cells with stimulatory and immunological properties. Many plasma-based devices do not use anticoagulants when harvesting blood, anticipating platelet activation and the release of clotting factors, including thrombin, to convert fibrinogen into a fibrin matrix. Fibrin polymerization, often induced by calcium chloride, results in a denser fibrin matrix compared to non-activated PRP. Following therapeutic application, fibrinolysis will liberate fibrin matrix components for a prolonged period, unlike activated PRP clots that lack excessive plasma fibrin and other proteins.

### 5.2. Variables in PRP Formulation

The quality and quantity of specific PRP products, determined by platelet dosages and leukocyte subpopulation bioformulations, are directly related to high cellular recovery rates from a unit of whole blood after gravitational density separation, as seen in double-spin PRP devices [36]. Advanced PRP devices should be proficient in preparing different validated bioformulations to produce various platelet dosing and leukocyte populations and concentrations. It is, however, difficult to compare PRP outcome results accurately due to the wide variation in reported protocols for PRP preparation, including the total amount of whole blood donated, the type of anticoagulants used, device physical characteristics, the recovery rates of platelets and leukocytes, and centrifugal variances [37,38,39]. These variable preparation characteristics, responsible for the heterogeneity of PRP preparations in MSK applications, have been discussed in more detail by Cherian et al. [40]. PRP centrifuge performance variables, such as the number of spin cycles, centrifugation speed, duration per spin cycle, and acceleration and deceleration speeds, are rarely mentioned. Thus, the characteristics of the ultimately extracted orthobiological products are scarcely understood. Piao et al. identified critical factors to guarantee effective cellular yields in PRP orthobiologics, such as centrifuge acceleration profiles and spin time for the maximum recovery rate of platelets and leukocytes [39]. Other substantial factors that determine the total number of available platelets for dosing include the total pre-donated anticoagulated blood volume prior to PRP processing and the geometrical mathematics and physical properties of PRP devices used for cell concentration.

## 6. Defining the Biological Content of PRP and Platelets

**Figure 2:**
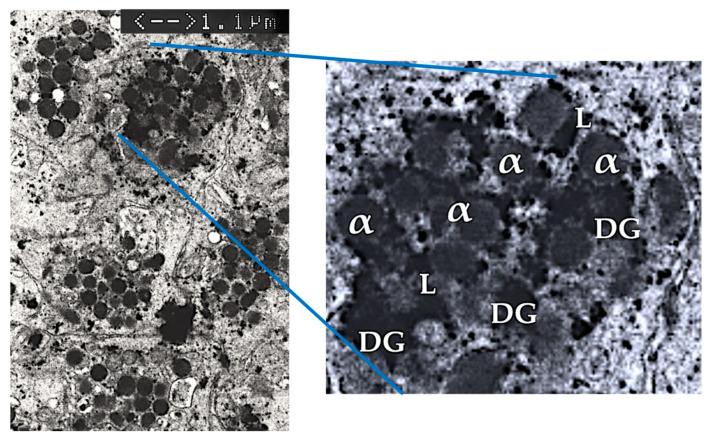
Electron microscopic image (JEOL-2000FX transmission electron microscope) of intact non-activated human PRP platelets (magnification ×7000). The blue lines mark a single platelet in a vial of PRP. The magnification of a single platelet reveals the three platelet granular structures. Adapted and modified from [45]. Abbreviations: ɑ: alpha-granules; DG: dense granules; L: lysosomes.

On the outer surface of platelets, glycoprotein receptors and adhesion molecules are present, which play crucial roles in platelet activation and aggregation [15]. Inside the platelet, there are three distinct structures: alpha-granules, dense granules, and lysosomes, as demonstrated in Figure 2. Additionally, and less known, are platelet-derived exosomes, carrying many molecules that are transported to treated tissue sites [41].

### 6.1. Platelet ɑ-Granules

The ɑ-granules of platelets are the most frequently cited intraplatelet structures due to their rich content of many platelet growth factors (PGFs), including platelet-derived growth factor (PDGF), vascular endothelial growth factor (VEGF), connective tissue growth factor (CTGF), basic fibroblast growth factor (b-FGF), epidermal growth factor (EGF), and transforming growth factor-b (TGF-b) [15,42]. To a lesser extent, hepatocyte growth factor (HGF), and insulin-like growth factor-1 (IGF-1) are secreted after platelet activation [43]. Less frequently mentioned platelet ɑ-granules include platelet coagulation factors, cytokines, chemokines, and pro- and anti-angiogenetic regulators. These specific cytokines and chemokines serve various functions, including modulating inflammation, promoting cell migration and proliferation, and regulating angiogenesis, all of which are essential for tissue repair and regeneration.

### 6.2. Platelet Dense Granules

The dense granules of platelets contain serotonin, adenosine diphosphate (ADP), polyphosphates, histamine, and epinephrine [24]. These substances primarily modulate platelet activation and thrombus formation. Importantly, many of these elements also have immune cell-modifying effects. For instance, platelet ADP is recognized by dendritic cells (DCs), leading to an increase in antigen endocytosis. DCs are crucial for initiating T-cell immune responses and governing the protective immune response, linking the innate and adaptive immune systems via inflammatory T helper cells (Th-2 cells) [44]. Additionally, platelet serotonin induces T-cell migration and promotes the differentiation of monocytes into DCs. In PRP, these dense granule-derived immune modifiers are highly enriched and exert substantial immune regulatory effects. This highlights the significant role of platelets in not only hemostasis but also in modulating immune responses and contributing to the overall inflammatory and immune regulatory processes within the body.

### 6.3. Platelet Lysosomal Granules

Several human in vivo studies have demonstrated ɑ- and dense granule secretion following platelet activation. However, there is limited data on the in vivo release of lysosomal content, which contains an array of acid hydrolases. In general, lysosomal functions have not been extensively studied, although Heijnen and van der Sluis highlighted their role as contributors to the cell’s digestive system, destroying substances from outside the cell and digesting old cytosolic components [46]. Furthermore, lysosomal activities are involved in extracellular functions such as fibrinolysis, vasculature remodeling, and the degradation of extracellular matrix components. Interestingly, lysosomes contain proteases and cationic proteins with bactericidal activity and are interconnected with macrophages in phagocytosis [47]. This suggests that lysosomes may also participate in immune responses by aiding in the destruction of pathogens. Overall, the diverse functions of lysosomes underscore their importance in both intracellular and extracellular processes, contributing to cellular homeostasis and tissue remodeling.

### 6.4. Platelet Exosomes

Exosomes are nanosized extracellular vesicles (EVs) that originate from intracytoplasmic multivesicular bodies (MVBs) and are directly released into the extracellular space upon the fusion of the MVB membrane with the plasma membrane [48]. Platelet-derived exosomes (PLT-EXOs) are stored in platelet ɑ-granules, and they are released from the platelets through extracellular secretion [49]. EVs, and thus PLT-EXOs, facilitate intracellular communication between (long-distance) cells in the body [50].

## 7. Identifying and Comprehending HD-PRP Characteristics

In two major device comparison studies, Fadadu and Magalon found that the platelet concentration was significantly lower in plasma-based PRP devices [36,51]. They utilized a low whole-blood volume and a single-spin centrifugation method, in contrast to a larger whole-blood volume and double-spin centrifugation method, to acquire a highly concentrated PRP product (HD-PRP) with significantly higher PGF concentrations. Everts et al. demonstrated that tissue repair, regeneration, and wound healing capacities are lower using plasma-based devices when compared to HD-PRP and buffy-coat PRP systems [42]. Notably, some plasma-based PRF devices have over 20 different sub-formulations and preparation protocols, varying the centrifugal force, speed, and processing time [35].

### 7.1. Platelet Dose

Clinicians should consider adopting the concept of total deliverable platelets (TDP) as an improved PRP quality parameter, as it indicates the number of platelets accurately delivered to a single treatment site. Our group presented for the first time the rationale and feasibility of implementing TDP dosing strategies, including biological formulations, to treat different pathoanatomic pathologies [24]. This new quality treatment standard is supported by the translation of in vitro data to clinical practice. Giusti et al. clearly demonstrated the need to deliver 1.5 billion platelets/mL to induce a significant angiogenic response [52].

Further studies are warranted to determine the optimal TDP dosing for different soft tissue types and pathologies, considering the chronicity of the disorder (acute vs. chronic). Similarly, Berger et al. indicated that higher platelet concentrations, prepared from platelet lysate preparations, resulted in more tenocyte proliferation and migration in a dose-dependent manner [53]. More recently, Bansal and associates injected 10 billion platelets into patients diagnosed with knee osteoarthritis and observed a consistent clinical effect regarding pain, inflammatory markers, and function after a single injection over a 12-month period [54].

### 7.2. Leukocytes in PRP

In the literature, PRP preparation protocols are generally underreported, and most studies do not include detailed PRP preparation methods, with inconsistent laboratory data on platelet counts, platelet dose, and specific leukocyte population concentrations [55]. This lack of reporting is surprising because PRP preparations contain varying leukocyte populations and concentrations, which can significantly impact inflammation, immunomodulation, nociceptive effects, tissue repair, and regeneration.

Currently, the use of quality hemocytometry to document and track the effects of PRP preparations is becoming increasingly common in clinical settings.

Different PRP preparation devices produce varying leukocyte counts and populations, with dissimilar neutrophil, lymphocyte, and monocyte cell concentrations and ratios in the final PRP preparation [56,57]. Plasma test tube-based PRP devices produce very few leukocytes and significantly fewer platelets compared to more advanced PRP devices. Sophisticated PRP devices produce a buffy-coat stratum where leukocytes are significantly concentrated, except for eosinophils and basophils, the cell membranes of which are too fragile to withstand centrifugal processing forces.

Physicians should carefully consider the inclusion of leukocytes in HD-PRP bioformulations, as these cells influence the intrinsic biology of both acute and chronic tissue lesions due to their immune and host-defense mechanisms. The presence of specific leukocyte populations in HD-PRP preparations will likely lead to significant differences in cellular and tissue effects. Considerations regarding bioformulations should be based on the specific musculoskeletal pathology being treated, the cellular properties of tissue structures, and the chronicity of the tissue pathology.

#### Platelet–Leukocyte Interactions in HD-PRP

Leukocytes in PRP preparations can be included or avoided, with neutrophils raising concern due to their pro-inflammatory cytokine activities and the release of MMPs, potentially escalating any early onset inflammatory response in injured hard and soft tissues [57]. However, their role in resolving inflammation is also significant [58]. Unfortunately, the PRP literature has primarily focused on individual leukocytic cells, like neutrophils, and their functions rather than the combined platelet–leukocyte interactions, including the presence of monocytes and lymphocytes. Activated HD-PRP platelets release lipid mediators that modulate inflammation [59]. Activated platelets can convert arachidonic acid to anti-inflammatory lipoxins, preventing further neutrophil recruitment and promoting tissue healing [60]. HD-PRP bioformulations with high platelet and monocyte yields mimic Th-2 cell activity, producing anti-inflammatory interleukin (IL) 4 [61]. Additionally, HD-PRP platelets can polarize macrophage subtypes, thereby modulating immune cell interactions [62], stimulating complex cell–cell interactions in immunomodulatory mechanisms, like in osteoarthritis and tendinopathies [63].

Recently, the application of HD-PRP containing a full leukocyte buffy coat for soft tissue pathologies has shown benefits, without significant adverse events [64,65].

### 7.3. Platelet-Derived EVs (PLT-EVs) and Exosomes (EXOs)

EVs are involved in various biological processes, including immune response, antigen presentation, cell migration, and tissue regeneration [66]. They can transfer molecules from one cell to another, influencing the recipient cell’s behavior and function. EVs are membrane-bound vesicles with a lipid bilayer that are secreted by almost all types of cells. These vesicles play vital roles in the human body, serving as crucial mediators for intercellular communication [48]. Based on size, biogenesis, and secretion mechanism, EVs are divided into three categories: EXOs, microvesicles, and apoptotic bodies [67].

The most common type of EVs in circulation are PLT-EVs, released upon the activation of platelets by various factors, with a size varying from 100 nm to 1 micrometer [68]. PLT-EVs have capabilities comparable to platelets and are thought to impact various biological processes such as coagulation, wound healing, and inflammation. They can stimulate cellular differentiation, improving musculoskeletal or neurological regeneration [69,70]. Unlike platelets, PLT-EVs can pass through all natural tissue barriers and deliver their cargo to remote tissue sites [71,72], extending their regenerative capabilities beyond the blood.

Exosomes are a type of EV and are formed within MVBs inside cells, where they bud off as intraluminal vesicles (ILVs) before being released into the extracellular space when the MVB fuses with the plasma membrane [73]. Exosomes range in size from 50 to 150 nm. Furthermore, EXOs and PLT-EXOs have no obvious adverse effects such as immunogenicity or tumorigenicity [74,75].

#### Autologous PLT-EXOs

**Figure 3:**
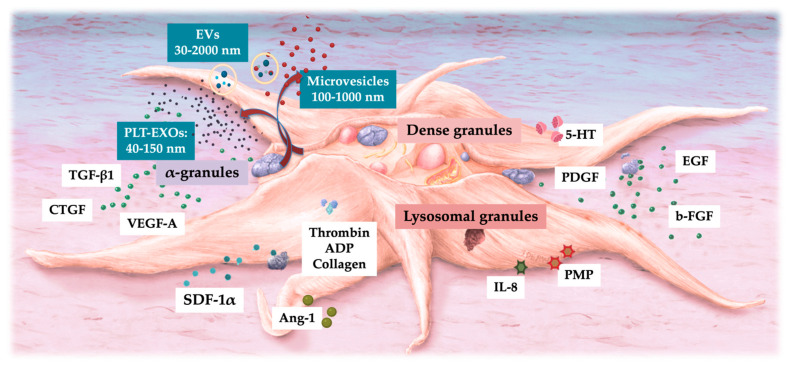
Graphical representation of an activated platelet releasing its granular content. Agonists like thrombin, ADP, and collagen activate non-activated platelets in HD-PRP. As a result, all three types of platelet granules release their stored molecules, such as growth factors, chemokines, cytokines, adhesion proteins, and EVs, along with the cargo contained within the platelet microvesicles and exosomes. Adapted and modified from Everts et al. [42]. Abbreviations: PLT-EXOs: platelet exosomes; EV: extracellular vesicle; TGF: transforming growth factor; VEGF: vascular endothelial growth factor; SDF: stromal cell-derived factor; ADP: adenosine phosphate; 5-HT: serotonin; PGDF: platelet-derived growth factor; EFG: epidermal growth factor; b-FGF: basic fibroblast growth factor; CTGF: connective tissue growth factor; PMP: platelet microparticles; IL-8: interleukin-8; Ang: angiopoietin.

PLT-EVs, including PLT-EXOs and MSC-derived exosomes (MSC-EXOs), have gained significant clinical and research interest to treat a variety of clinical pathologies, acting as drug delivery vehicles and diagnostic indicators [68]. These vesicles are valued for their potential in therapeutic applications and as diagnostic tools due to their ability to transport bioactive molecules and facilitate intercellular communication [76]. PLT-EVs are an integrated part of platelets, as visualized in Figure 3. They can support coagulation and angiogenesis, regulate immunity, and accelerate tissue repair [50].

Clinically, PLT-EXOs are beneficial for treating chronic injuries and trauma, alleviating knee osteoarthritis, and promoting wound healing. Torreggiani et al. isolated exosomes from PRP as novel effectors in human platelet activity and discussed the use of PLT-EXOs for bone tissue regeneration [77]. Recent studies further emphasize the beneficial effects of PLT-EXOs from PRP in preventing osteonecrosis and promoting the re-epithelialization of chronic wounds [78].

While PLT-EXO technologies have significant potential, the US-FDA restricts the clinical application of allogeneic PLT-EXOs. Currently, only very limited technologies are available for the manufacturing of autologous PLT-EXOs and for their use in clinical settings. Briefly, autologous patient pure exosomes (PPX™) (Zeo Scientifix, Nova Southeastern University, Fort Lauderdale, FL 33328, USA) are manufactured from a fresh unit of anticoagulated patient whole blood. Then, a double-spin PRP preparation is subjected to an innovative proprietary production process to extract PLT-EVs and PLT-EXOs, generating a high acellular concentration of autologous PLT-EXOs (400 billion exosomes per vial). The entire procedure is performed in an US-FDA-registered and cGMP-compliant laboratory in accordance with stringent safety and efficacy standards.

### 7.4. Immunomodulation

In both acute and chronic conditions, injured and degenerated tissues, as well as foreign bodies, are rapidly identified. This recognition initiates inflammatory pathways and is related to the start of the wound healing cascade. The immune response involves both the innate and adaptive immune systems, with leukocytes playing essential roles in both systems and overlapping between the two [15]. Monocytes, macrophages of phenotypes 1 and 2, neutrophils, dendritic cells, and natural killer cells perform fundamental tasks in the innate system, nonspecifically identifying intruding microbes or tissue fragments and stimulating their clearance [79]. Lymphocytes and their subsets have similar capacities in the adaptive immune system. Under homeostatic circumstances, platelets are among the first cells to identify endothelial tissue injury and detect the presence of microbial pathogens. Following HD-PRP injections, high concentrations of platelets accumulate and aggregate at treated tissue sites. Following platelet activation, abundant platelet agonists and biomolecules are released, further enhancing platelet activation while expressing platelet chemokine receptors. Under normal circumstances, this results in the rapid accumulation of peripheral platelets at the site of injury or infection. Subsequently, neutrophils, monocytes, and dendritic cells are recruited for an early phase immune response [80]. As HD-PRP therapies provide inflammatory stimuli, platelet receptors change their surface expression to stimulate platelet–leukocyte interactions by forming platelet–leukocyte aggregates to regulate inflammation and ultimately tissue repair. More precisely, neutrophils and monocytes are both active participants in these aggregate formations, thereby contributing to the innate immune response [81]. This coordinated response ensures that the immune system can effectively identify and respond to injuries and infections, promoting healing and tissue repair.

### 7.5. Angiogenesis

Angiogenesis, the restoration of blood vasculature and subsequent tissue perfusion, plays a crucial role in wound healing and tissue regeneration This process is particularly stimulated by high concentrations of VEGF, PDGF, and b-FGF, which are abundantly present in HD-PRP [42]. VEGF is the most important and widely studied angiogenic factor, produced in large amounts by platelets, keratinocytes, macrophages, endothelial cells, and fibroblasts during angiogenesis and classical wound healing.

Tissue damage, cell disruption, chronic inflammatory conditions, and hypoxia are strong inducers of angiogenic factors, including several types of VEGF and their typical receptors. Specifically, VEGF-A promotes the initial phase of angiogenesis and is important for wound healing. It binds to the dedicated VEGF-1 and VEGF-2 receptors, initiating blood vessel organization, chemotaxis, proliferation, and endothelial cell differentiation, respectively [82]. These coordinated responses ensure that new blood vessels form efficiently, providing the necessary oxygen and nutrients to support the wound healing process and tissue regenerative pathways.

### 7.6. Nociception

In 2008, Everts et al. were the first to report on the analgesic effects of an activated PRP formulation, and they observed a significant reduction in pain and opioid use, as well as more successful post-surgical rehabilitation [83]. Various clinical studies have demonstrated significant pain reduction or elimination after PRP treatments [84,85]. However, others reported little to no pain relief. TDP, platelet dosing, and PRP bioformulations have been identified as key features contributing to consistent painkilling effects [42]. Additional variables affecting pain modulation that have been cited are the type of injury, treated tissue types, PRP application techniques, and the use of platelet activators [86].

Kuffler studied the use of PRP to alleviate chronic neuropathic pain due to damaged non-regenerated nerves and demonstrated that neuropathic pain relief was noted within three weeks, and pain was eliminated or significantly reduced for more than six years [87]. Similar painkilling effects were observed by Mohammadi et al. in post-surgical wound care patients, with a higher incidence of angiogenesis in patients treated with PRP [88]. They concluded that triggering neo-angiogenesis was necessary to optimize tissue oxygenation and nutrient delivery.

The optimal PRP platelet dose and bioformulation for maximal pain relief are currently unknown. Published data suggest that PRP should contain at least 1.0 billion platelets per milliliter to provoke painkilling effects, with higher platelet doses leading to better outcomes and higher patient satisfaction [89]. The exact mechanisms behind this dose-dependent effect are still under investigation, highlighting the need to optimize PRP formulations for the best therapeutic results. Further research is needed to establish the precise platelet concentration and bioformulation for effective pain relief in different clinical scenarios.

## 8. Aspects of Adipose Tissue Biology

Currently, innovative strategies are being developed to repair soft tissue pathologies, select cartilage defects, alleviate pain, improve wound healing, and ultimately achieve functional tissue repair. In addition to a variety of adipose tissue preparations such as t-SVF, which are frequently used for regenerative applications in plastic reconstructive and cosmetic surgical procedures, other biological preparations have also gained prominence. These include PRP, platelet lysate (PL), BMAC, autologous PLT-EXOs, and non-autologous Wharton’s jelly. These preparations have become increasingly popular in the fields of non-surgical sports medicine and orthopedics, as evidenced by numerous studies and clinical applications [90,91,92].

### 8.1. Adipose t-SVF Hallmarks

Adipose tissue preparations have been successfully used in minimally invasive and non-surgical reconstructive procedures and in guided injections into damaged, degenerative, or non-healing wounds. The rationale for using t-SVF is that it is a heterogeneous ABP prepared from highly vascularized and stable connective tissue due to cell-to-cell or cell-to-matrix connections, which are considered a prerequisite for such stem cells to send and receive signals [93]. Adipose tissue, with its important bioactive scaffolding capabilities, should therefore be recognized as a multifaceted microvascular organ in the form of lipoaspirates, consisting of various cellular tissue and stromal elements, including AD-MSCs [94]. Furthermore, concentrated and compressed t-SVF provides a physiological multicellular scaffold containing AD-MSCs along with other stromal vascular cells. Minimally invasive access techniques are employed to harvest from several subcutaneous fat deposits, including the abdominal, perigluteal, thigh, and flank areas [95]. During the procedure, a subcutaneous injection of a dilute local anesthetic solution (tumescent) is administered to numb the area of adipose harvesting and facilitate the creation of a t-SVF suspension [96]. After a brief waiting period, a blunt-tipped, coated atraumatic microcannula is introduced through a small puncture using extensive pre-tunneling passages in the skin and attached to a closed syringe for manually controlled, low-negative-pressure lipoaspiration. A correctly performed lipoaspiration procedure preserves the neurovascular structures at the donor site with a minimal level of discomfort for the patient. Furthermore, the cell yield and viability of AD-MSCs are rarely impacted by liposuction techniques [97].

**Figure 4:**
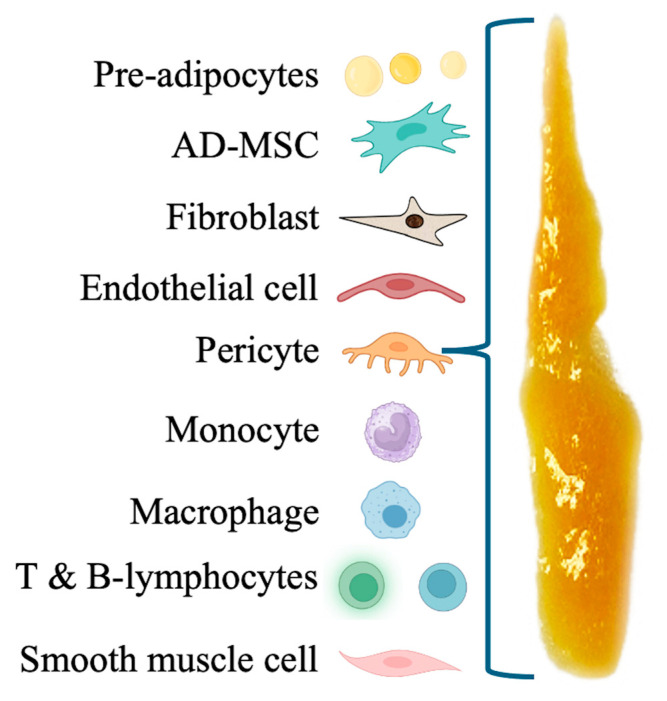
Graphic representation of t-SVF cellular content. t-SVF comprises a heterogenous cell population extracted from adipose tissue, containing a mix of different cell types, including highly proliferative adipose stem/progenitor cells.

Following lipoaspirate centrifugation, t-SVF-based preparations can be accessed, consisting of cellular and matrix components derived from adipose tissue. These preparations are used in advanced regenerative medicine procedures. SVF is a complex mixture of various cell types, including endothelial cells, immune cells, fibroblasts, macrophages, pericytes, and a significant population of AD-MSCs [98], as depicted in Figure 4. Embedded in and attached to collagen fibers and other components of the extracellular matrix, these cells contribute to the regenerative properties of the SVF [7].

**Figure 5:**
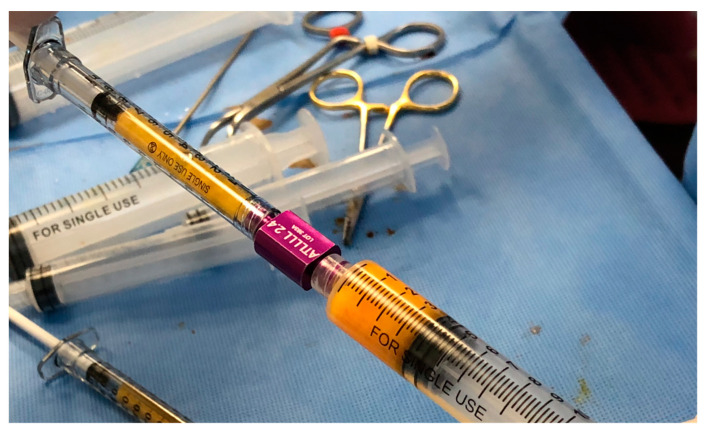
Mechanical emulsification using a 2.4 mm restraining adaptor (Tulip^®^ Medical, San Diego CA, USA).

Prior to regenerative applications, various SVF preparation protocols are available to prepare either fully emulsified nanofat in small aggregates (t-SVF) or cellular SVF (c-SVF), including a preparation method based on enzymatic digestion techniques using collagenase [7,99]. t-SVF preparations are created by mechanical emulsification, reducing the size of macrofat to either partially or fully emulsified aggregates, known as partially emulsified fat or nanofat, as shown in Figure 5.

Importantly, the fully emulsified nanosized t-SVF aggregates present an intact microenvironment, whereby the fully emulsified small nanofat aggregates are essentially devoid of adult adipocytes. Generally, nanofat t-SVF preparations are considered the most potent and valuable form of t-SVF for regenerative and wound healing purposes [99].

Freshly prepared t-SVF can be directly administered to the patient without the need for cell expansion techniques or additional cell separation preparations. Unlike cultured MSCs, emulsified t-SVF is not a 100% acellular product, as it contains both cellular fragments and important native adipose structural components. An advantage of t-SVF compared to cultured acellular MSCs is that it offers the ability to provide a bioactive cellular tissue scaffold [94].

### 8.2. t-SVF Components

The application of t-SVF in wound healing, tissue repair, and regenerative therapies is predicated on its rich content of regenerative cells and factors. SVF cells possess the capacity to differentiate into various cell types, engage in cell-to-cell communication through paracrine signaling, and possibly contribute to secreting ECM components [100]. These attributes provide crucial elements in response to damaged or degenerative cells. The presence of typical heterogeneous SVF-based cells can be assessed using enzymatic methods and quantified with advanced laboratory flow cytometry techniques, ensuring the quality and potential effectiveness of both t-SVF and c-SVF in therapeutic applications. AD-MSCs are the most prominent stem cells in t-SVF, with the potential to differentiate into various cell types, including osteoblasts, adipocytes, chondrocytes, tenocytes, and myocytes. MSCs, with high self-renewal, proliferation, and differentiation potential, can be derived from various sources, including the skeletal muscle, synovium, and periosteum [101].

#### 8.2.1. AD-MSCs

Adult MSCs are undifferentiated and multipotent cells that are found in virtually every organ of the body and are capable of self-renewal [4]. The most common sources of ubiquitous MSCs are subdermal adipose tissue and bone marrow [102]. In bone marrow, MSCs represent a small fraction, ranging from 0.001% to 0.01% of nonhematopoietic, multipotent MSCs [103]. MSCs have the ability to differentiate into various cells of mesodermal origin, including adipocytes, chondrocytes, myocytes, and osteoblasts, when exposed to specific signaling pathways [104]. In vitro, these same cells have been shown to develop into neuroectodermal lines, thereby acting in a pluripotential capacity [105]. In addition to their potential to promote immune modulation, angiogenic activity, anti-inflammatory activity, and anti-apoptotic activity, they also release numerous cytokines and growth factors that confer immunomodulatory, anti-apoptotic, and anti-inflammatory effects on many heterogeneous populations of cells [10].

AD-MSCs and BM-MSCs share many the same biological characteristics and transcriptome profiles [106]. However, some differences in their immunophenotypes, differentiation potential, proteomes, and immunomodulatory activities have been reported.

Adipose tissue, on the other hand, has been reported to contain larger quantities of mesenchymal and progenitor cells. Due to this higher concentration of reparative and regenerative cells, adipose-derived cell therapies have become increasingly popular as a treatment option, surpassing the effectiveness of BMAC- and peripheral blood-derived preparations [107]. Furthermore, AD-MSCs are preferred over bone marrow-derived counterparts due to their superior accessibility, higher proliferative and repair capacities, and lower donor site morbidity [108]. AD-MSCs exert paracrine, anti-inflammatory, and immunomodulatory effects depending on environmental conditions [109,110]. Their therapeutic effects are likely mediated by paracrine signaling and the release of growth factors and cytokines that influence the intra-articular environment [111]. Furthermore, AD-MSCs are known to polarize M0 macrophages and dendritic cells to an anti-inflammatory phenotype [112]. AD-MSC preparations have shown excellent safety profiles and promising clinical efficacy in treating soft tissue and musculoskeletal disorders like knee and shoulder pathologies [113].

#### 8.2.2. Pericyte Stem Cells (PSCs)

Many researchers and clinicians believe that pericyte stem cells (PSCs) are ubiquitous cells, representing the original “stem” cell originator, and are fully capable of all the functions of MSCs [114]. PSCs are located on the walls of capillaries in perivascular and paravascular locations and work intimately with endothelial and intra-adventitial cells [115]. PSCs also possess pluripotent capabilities, including the ability to stabilize blood vessels and to differentiate into other types of cells, including smooth muscle cells, adipocytes, osteoblasts, chondrocytes, and even neural lineages, contributing significantly to tissue regeneration, repair, and wound healing [116].

#### 8.2.3. Endothelial Cells

Endothelial cells, which line blood vessels, play key roles in forming new blood vessels through angiogenesis [117]. t-SVF aids in the promotion of angiogenesis, as endothelial cells and growth factors promote the formation of new blood vessels, enhancing oxygen and nutrient supply to the wound or damaged tissue, thereby supporting tissue healing [118].

#### 8.2.4. Fibroblasts

Fibroblasts in t-SVF contribute to the synthesis of collagen and other extracellular matrix components, which are essential for the structural integrity and function of repaired tissues [119]. This comprehensive approach ensures that t-SVF can effectively support the healing and regeneration processes in various types of tissue damage. Fibroblasts hold crucial roles in wound closure and the strength of healed tissues [120].

#### 8.2.5. Immune Cells

Various immune cells, originating from hematopoietic cell lineages (e.g., monocytes, lymphocytes, and various macrophage phenotypes), are involved in modulating the immune response. Immune cells and anti-inflammatory cytokines are present in t-SVF, playing a crucial role in modulating the body’s immune response [121]. The primary immune cells associated with anti-inflammatory activities are M2 macrophages and regulatory T cells (Tregs) [122]. These cells secrete the anti-inflammatory cytokine IL-10, reducing chronic inflammation. Additionally, T helper cells, or Th cells, more specifically Th2 cells, produce TGF-β and IL-4, further supporting the anti-inflammatory environment [123]. Together, these cells and cytokines modulate the body’s immune response, reducing inflammation and promoting a more conducive environment for tissue repair and tissue regeneration [124,125].

## 9. The Regenerative Marriage Between HD-PRP and Adipose Tissue

**Figure 6:**
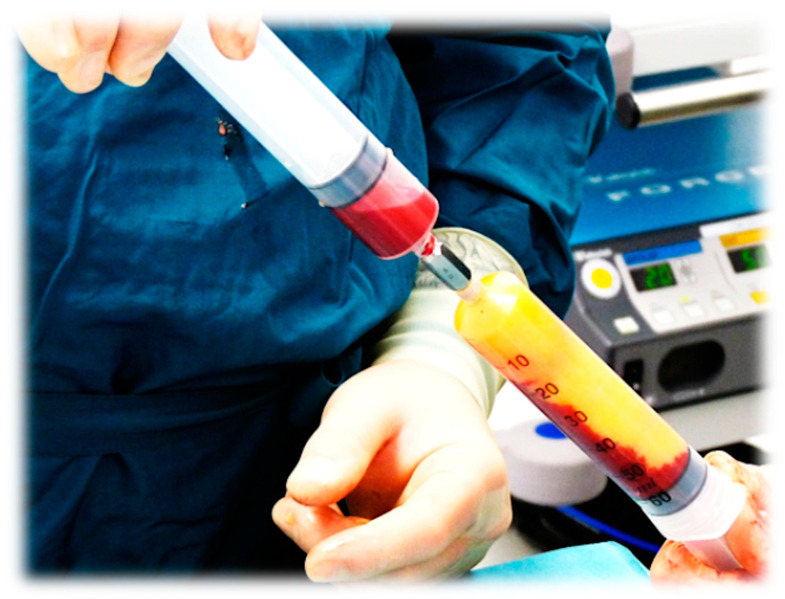
Mixing a high volume of HD-PRP (leukocyte-rich preparation) with a high volume of partially emulsified adipose tissue for a plastic reconstructive procedure.

**Figure 7:**
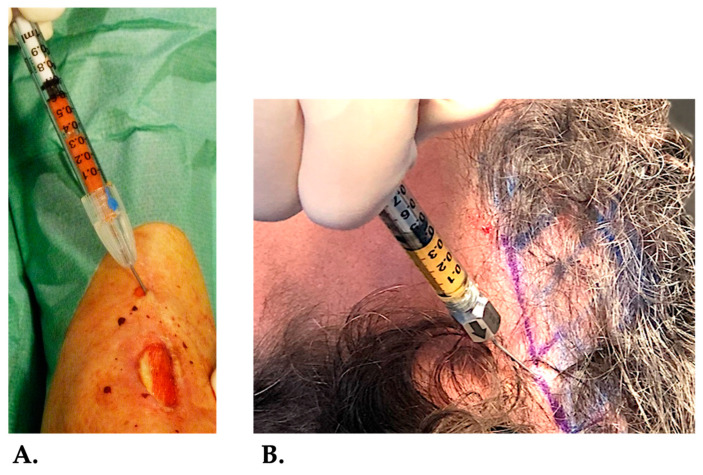
HD-PRP mixed with t-SVF serving as a biocellular graft. In (**A**), the combined biocellular graft is injected in the wound edges of a chronic venous lower extremity ulcer. In (**B**), the HD-PRP and t-SVF mixture is injected in the scalp to stimulate hair growth in a patient suffering from alopecia errata.

The efficacy of PRP and t-SVF as biological treatment options has been extensively studied. The combination of PRP and t-SVF (Figure 6) has been utilized as an innovative autologous biological multicellular treatment platform, mostly employed in aesthetic and plastic reconstructive surgeries, as well as for treating non-healing chronic wounds Figure 7). The synergistic effects of PRP and t-SVF enhance tissue regeneration and repair, offering a comprehensive and effective solution for various medical and cosmetic needs.

### 9.1. Rationale for Compounding HD-PRP with t-SVF

Tissue repair, regeneration, and wound healing are complex biological processes aimed at restoring the integrity and function of damaged or degenerated tissues. These restorative processes follow well-defined cascades of events, involving numerous systemic and local cellular activities occurring sequentially.

HD-PRP and t-SVF biological technologies are designed to mimic the initiation of classical and angiogenesis healing cascades in various medical indications, including cosmetic and plastic reconstructive surgical and non-surgical procedures [126]. Among other benefits, compounding HD-PRP and t-SVF is thought to potentially enhance fat graft survival, potentially avoiding fat necrosis, and to reduce inflammation by promoting improved vascularization and providing anti-inflammatory signals at the graft site, thereby improving the overall healing process and stimulating the recipient tissue site [127,128]. Notably, Yoshimura et al. clearly showed limited adipocyte survival in transplanted fat grafting treatments [129]. They presented the concept of the “cell survival theory”, in which transplanted adipocytes partly survive in response to microenvironmental changes such as tissue ischemia and applied mechanical forces, leading to ischemic adipocyte cell death [130,131]. This results in nanofat small aggregate utilization in aesthetics and orthobiological applications because nanofat leads to better adipocyte survival when compared to traditional fat grafting techniques due to a high concentration of AD-MSCs and other SVF constituents [132].

### 9.2. Synergistic Effects

Combining PRP and t-SVF has demonstrated notable effects on the metabolism of AD-MSCs. Additionally, it has promoted parenchymal cell proliferation and facilitated immunomodulation, impacting both the transplanted component and the damaged or aging recipient sites through various biological pathways [133,134].

The synergy of PRP and AD-MSCs has been demonstrated to significantly stimulate tissue vascularization in wounds compared to PRP or AD-MSCs alone [133]. It is believed that PRP enhances the angiogenic potential of AD-MSCs by activating the MSC secretome, resulting in the increased synthesis of VEGF and stromal cell-derived factor 1, which consequently improves blood vessel formation and endothelial cell migration [135].

#### 9.2.1. HD-PRP and t-SVF Matrix Formation

Additional synergistic effects between t-SVF and a PRP-induced fibrin matrix have been observed regarding the secretion of high VEGF concentrations when both biologics are combined [136].

This matrix serves as a cellular scaffold, retaining concentrated platelet elements, leukocytes, t-SVF cells, cytokines, and other proteins for an extended period, functioning as a sustained, extended-release matrix [6,137]. Furthermore, this scaffold inhibits the apoptosis of adipocytes. Interestingly, the combination of PRP and AD-MSCs induced an increase in mitochondrial respiration and oxygen consumption, resulting in elevated adenosine triphosphate production [126].

#### 9.2.2. Optimizing Cell Migration

Cell migration is a fundamental, and underestimated, process in all multicellular organisms, playing a critical role not only during development but also throughout an organism’s life [138]. This process is particularly important during wound healing, where cells must move to the site of injury to facilitate repair and wound closure, as well as the restoration of normal tissue architecture [139]. Cell migration is directed by cell signaling or extracellular cues like soluble factors, cell-bound ligands, or ECM components, eliciting a wide range of intracellular responses [140]. These cues can affect EV transportation pathways over long distances, which are essential for transporting cargo within cells and steering it to the correct location. Correspondingly, cues receive signals from neighboring cells and the extracellular matrix, vital for maintaining cellular function. Hence, cues influence gene transcription and protein production to further support the integrity of cell migration and further propagate migratory signals within the cell [141].

## 10. Conclusions

HD-PRP and t-SVF are powerful, high-yield cellular biological products that can be safely prepared at the point of care, either separately or as a combined biological product, to repair and regenerate tissues, aiming for functional restoration. HD-PRP exhibits unique cellular and acellular components, making it a complex biological preparation, containing many platelet-derived growth factors, cytokines, chemokines, PLT-EXOs, proteins, and leukocytes depending on the executed preparation protocol. t-SVF, a heterogenous mixture of cells obtained from adipose tissue, includes AD-MSCs and their progenitor cells, endothelial cells, pericytes, and a variety of immune cells. Both autologous preparations can be employed in non-surgical and bio-surgical procedures, including wound healing treatments, to improve patient outcomes and decrease complications.

Combining HD-PRP with t-SVF is considered beneficial because it creates multiple synergistic effects, which are capable of enhancing angiogenesis, increasing cell proliferation, modulating the immune response, and providing a supportive extracellular matrix.

This comprehensive combined multicellular approach is a promising strategy for enhancing tissue healing and regeneration. This biological preparation can create an optimal environment for tissue repair and regeneration, leading to improved clinical outcomes in patients suffering from recalcitrant and chronic wounds and providing support in skin rejuvenation and soft tissue repair procedures (scar treatments) for various MSK disorders like osteoarthritis and tendinopathies.

Lastly, more clinical and translational studies are required to further elucidate the biological potential of these preparations and further demonstrate their efficacy.

## Data Availability

No new data were created or analyzed in this study.

## Abbreviations

The following abbreviations are used in this manuscript:

ABP	autologous biological preparation
AD-MSC	adipose-derived mesenchymal stem cell
ADP	adenosine diphosphate
b-FGF	basic fibroblast growth factor
BMAC	bone marrow aspirate concentrate
CTGF	connective tissue growth factor
DC	dendritic cell
ECM	extracellular matrix
EGF	epidermal growth factor
EV	extracellular vesicle
HD-PRP	high-density platelet-rich plasma
HGF	hepatocyte growth factor
IGF-1	insulin-like growth factor-1
IL	interleukin
ILV	intraluminal vesicle
LP-PRP	leukocyte-poor PRP
LR-PRP	leukocyte-rich PRP
MSC	mesenchymal stem cell
MSK	musculoskeletal
MVB	multivesicular body
PDGF	platelet-derived growth factor
PGF	platelet growth factor
PLT-EXO	platelet-derived exosome
PPX	patient pure exosome
PRF	platelet-rich fibrin
PRP	platelet-rich plasma
PSC	pericyte stem cell
SVF	stromal vascular fraction
t-SVF	tissue stromal vascular fraction
TDP	total deliverable platelets
TGF-b	transforming growth factor-b
Th-cell	T helper cell
Treg	regulatory T cell
VEGF	vascular endothelial growth factor
